# Trends in US Pediatric Asthma Hospitalizations, by Race and Ethnicity, 2012–2020

**DOI:** 10.5888/pcd21.240049

**Published:** 2024-09-19

**Authors:** Sophie Binney, W. Dana Flanders, Kanta Sircar, Osatohamwen Idubor

**Affiliations:** 1 Oak Ridge Institute for Science and Education, Oak Ridge, Tennessee; 2 Asthma and Air Quality Branch, Division of Environmental Health Science and Practice, National Center for Environmental Health, Centers for Disease Control and Prevention, Atlanta, Georgia; 3 Department of Epidemiology, Rollins School of Public Health, Emory University, Atlanta, Georgia; 4 US Public Health Service, Washington, DC

## Abstract

**Introduction:**

Some racial and ethnic minority communities have long faced a higher asthma burden than non-Hispanic White communities. Prior research on racial and ethnic pediatric asthma disparities found stable or increasing disparities, but more recent data allow for updated analysis of these trends.

**Methods:**

Using 2012–2020 National Inpatient Sample data, we estimated the number of pediatric asthma hospitalizations by sex, age, and race and ethnicity. We converted these estimates into rates using data from the US Census Bureau and then conducted meta-regression to assess changes over time. Because the analysis spanned a 2015 change in diagnostic coding, we performed separate analyses for periods before and after the change. We also excluded 2020 data from the regression analysis.

**Results:**

The number of pediatric asthma hospitalizations decreased over the analysis period. Non-Hispanic Black children had the highest prevalence (range, 9.8–36.7 hospitalizations per 10,000 children), whereas prevalence was lowest among non-Hispanic White children (range, 2.2–9.4 hospitalizations per 10,000 children). Although some evidence suggests that race-specific trends varied modestly across groups, results overall were consistent with a similar rate of decrease across all groups (2012–2015, slope = −0.83 [95% CI, −1.14 to −0.52]; 2016–2019, slope = −0.35 [95% CI, −0.58 to −0.12]).

**Conclusion:**

Non-Hispanic Black children remain disproportionately burdened by asthma-related hospitalizations. Although the prevalence of asthma hospitalization is decreasing among all racial and ethnic groups, the rates of decline are similar across groups. Therefore, previously identified disparities persist. Interventions that consider the specific needs of members of disproportionately affected groups may reduce these disparities.

**Summary**

**What is already known on this topic?**

Pediatric asthma outcomes vary by race and ethnicity. Some racial and ethnic minority communities have faced a disproportionate asthma burden for a long time.

**What is added by this report?**

Using estimates of pediatric asthma hospitalizations, we calculated rates and compared changes over time for 6 racial and ethnic groups. This analysis updates prior research and can inform asthma control strategies.

**What are the implications for public health practice?**

Although hospitalization rates are decreasing among all groups, declines are similar across groups, so previously identified disparities persist. Children from racial and ethnic minority groups, especially non-Hispanic Black children, remain disproportionately affected. Interventions considering the specific needs of members of these groups may be useful in addressing their high rate of asthma hospitalizations.

## Introduction

Asthma is a chronic condition in which a person’s airways become inflamed and narrow in the presence of certain triggers (1). Asthma affects approximately 1 in 15 children in the United States (4.7 million children in 2021) (2). Uncontrolled asthma can have severe, long-term consequences. Children with uncontrolled asthma have a lower quality of life (3) and may experience irrecoverable loss of lung function at an early age (4). Although no cure exists for asthma, proper management can lessen its effects and mitigate the risk of these adverse outcomes.

Racial and ethnic disparities exist in asthma prevalence, health care utilization, and mortality. The existence of such disparities has been known for decades, and they have persisted over time (5,6). A 2014 analysis of trends in racial disparities in childhood asthma outcomes in the US found that population-level disparities, as measured by the rate ratio, were either stable (emergency department [ED] visits and hospitalizations) or increasing (asthma attack prevalence and death) (6). However, this analysis was conducted on data only through 2010, and more recent data have been published.

We assessed the burden of asthma by using population-based rates of hospitalization, focusing on trends among children by racial and ethnic status from 2012 through 2020. Analyzing the most recent data from a nationally representative sample of hospitalizations, which has an improved sampling strategy and a more comprehensive means of data collection than that of prior research, provides an updated picture of the trends in racial and ethnic asthma disparities. Understanding the current state of racial and ethnic asthma outcome disparities will provide evidence that can be used to make informed decisions about future asthma control efforts.

## Methods

### Data sources

The primary data set we used for this analysis was discharge data from the National Inpatient Sample (NIS), Healthcare Cost and Utilization Project (HCUP), Agency for Healthcare Research and Quality (AHRQ) (7). The NIS is the largest publicly available, all-payer, inpatient health care database covering the US; it consists of a stratified sample of 20% of discharges from nonfederal, community hospitals, excluding rehabilitation and long-term acute care hospitals. The NIS contains data from approximately 7 million inpatient hospitalizations annually. To ensure a self-weighted sample, sampling rates vary for each stratum. More details about the NIS sampling strategy can be found on the HCUP User Support website (https://hcup-us.ahrq.gov/techassist.jsp). The NIS is sampled from the State Inpatient Databases, which participating state partners submit to AHRQ. NIS data sets are published annually, and we analyzed NIS data from 2012 through 2020, the most recent year for which data have been published.

We obtained estimates of the US population from the US Census Bureau Vintage 2020 Population Estimates (8,9). Every year, the US Census Bureau estimates the resident population for each year since the most recent decennial census using measures of population change. The Vintage 2020 estimates are based on the 2010 Census combined with vital and immigration records, without incorporation or consideration of the 2020 Census results (9). In this analysis, we treated these population denominators as counts, not estimates.

### Variables of interest

Our outcome of interest was asthma-related hospitalizations among children (aged 0–17 y). We identified asthma hospitalizations as those with a first-listed physician diagnosis beginning with 493 in the *International Classification of Diseases, Ninth Revision, Clinical Modification* (ICD-9-CM) (2012 to 2015) or diagnosis beginning with J45 in the *International Classification of Diseases, Tenth Revision, Clinical Modification* (ICD-10-CM) (2016 to 2020). This distinction was necessitated by a change in diagnostic coding procedures between the end of the third (September) and start of the fourth (October) calendar quarters of 2015. Patient age and sex are provided in the NIS. We categorized patient age into 3 groups (0–4 y, 5–9 y, and 10–17 y) on the basis of previous childhood asthma research (10). Patient sex was listed as either male or female.

We separated patient race and ethnicity into 6 categories: Hispanic, non-Hispanic White, non-Hispanic Black, non-Hispanic Asian, non-Hispanic Native American, and Other. The NIS and the US Census Bureau determine racial status in different ways. In the NIS, collection of race data varies by HCUP partner and may not be reported in the same manner across all facilities. The 2012–2020 NIS categorizes patients into 1 of 6 racial and ethnic categories (White, Black, Hispanic, Asian, Native American, and Other). In all HCUP data sets, Hispanic ethnicity takes precedence over race, meaning that when Hispanic ethnicity is documented separately from racial group, AHRQ classifies patients of Hispanic ethnicity as “Hispanic,” regardless of racial group identification. US Census Bureau data must adhere to the 1997 Office of Management and Budget standards, which stipulate that Hispanic ethnicity be determined separately from race (11).

In reconciling these differing methodologies, we opted to retain the HCUP-provided racial and ethnic categories and modify the population estimates. We used the estimated number of Hispanic people, regardless of racial status, as the denominator for that population. We then used the estimates of people identifying as non-Hispanic White alone, non-Hispanic Black alone, non-Hispanic Asian and Pacific Islander alone, and non-Hispanic Native American and Alaska Native alone as the denominators for their respective populations. We chose to group non-Hispanic Asians and non-Hispanic Pacific Islanders together based on the race-bridging methods used by the US Census Bureau (12). We used the number of people estimated to identify in the Vintage 2020 as 2 or more races as the denominator for the HCUP “Other” racial and ethnic group. We subsequently refer to the children in this numerator and denominator pair as “Other.”

### Statistical analysis

We used SAS version 9.4 (SAS Institute Inc) to weight the NIS data and generate estimates of the total number of pediatric asthma hospitalizations by sex, age group, and racial and ethnic group in each analysis year, as well as to determine the demographic makeup of patients in each year. Using weights for the NIS provided by AHRQ, we used survey procedures in SAS 9.4 to account for the complex survey design of the NIS. For each population subgroup, we then divided the weighted estimate of the number of hospitalizations for each year by the corresponding population denominator to estimate the population-based rates of hospitalizations by sex, age group, and racial and ethnic group. In reporting these rates, we separated the periods 2012–2015 and 2016–2019 because of potential shifts in diagnosis patterns due to the change in diagnostic coding. We also report rates for 2020 separately because the COVID-19 pandemic resulted in noticeable shifts in health care utilization, including a decrease in emergency medicine encounters among children with asthma after the onset of the COVID-19 pandemic (13,14).

To evaluate temporal trends, we used meta-regression (15,16) in which the dependent variable was the calculated hospitalization rate for each combination of race category and year. Key assumptions are correct model specification, independent observations, and normality of random effects (16). We specified the model to have a unique rate for each racial and ethnic group with a linear trend over years and a random effect of year. We assessed the key assumption of correctness of the model specification with residual plots and by considering more complicated models with interaction terms (year × race group [ie, a separate trend for each race group]) and higher order terms (year squared), as well as different random error terms (eg, random effect of race vs random effect of year). The Bayesian Information Criterion (BIC) was used to compare and select models (lower BIC preferred). We fit one model for the years 2012–2015 and a separate model for the years 2016–2019. The regression models included fixed effects for each of the 6 nonmissing race and ethnicity categories (indicator variables) and a linear term for year (continuous year variable). Children assigned a missing or invalid race and ethnicity status were excluded from this portion of the analysis. Analyses incorporated the known variance of the hospitalization counts due to sampling, and a random effect for each year. In sensitivity analyses, we evaluated a model in which each race category had its own trend and models with a random effect for race. Meta-regression analyses were conducted in R version 4.3.1 (17) and the package mixmeta, version 1.2.0 (16).

## Results

The total number of pediatric asthma hospitalizations decreased over both halves of the analysis (2012–2015 and 2016–2019, respectively), from 114,325 hospitalizations in 2012 to 87,065 in 2015, and from 80,235 in 2016 to 64,525 in 2019 (Table 1). A sharp decrease in total hospitalizations was seen between 2019 and 2020, with only 27,055 hospitalizations that year. Across all years, hospitalized patients were more frequently male (range, 59.4%–62.5%) and aged 0 to 4 years (range, 40.7%–48.9%). Non-Hispanic Black children made up the highest percentage of patients (range, 32.9%–36.2%), followed by non-Hispanic White children (27.7%–31.9%). Hispanic children made up between 20.1% and 23.3% of patients. Non-Hispanic Asian children represented around 3% of those hospitalized, while non-Hispanic Native American children made up less than 1% of patients. Children categorized in the Other racial and ethnic group represented between 4.9% and 6.2% of the sample each year. In every year, less than 7% of children had no listed race status (ie, missing/invalid), and this percentage was stable across years.

**Table 1:** Demographic Characteristics of Children^a^ Hospitalized for Asthma, US National Inpatient Sample, 2012–2020^b^

Characteristic	2012	2013	2014	2015^b^	2016	2017	2018	2019	2020
**Total no.**	114,325	100,765	103,260	87,065	80,235	75,905	74,295	64,525	27,055
**Sex, %**									
Male	62.5	61.9	62.3	61.3	60.9	61.5	60.8	61.0	59.4
Female	37.5	38.1	37.7	38.7	39.1	38.5	39.2	39.0	40.6
**Age, %, y**									
0–4	48.9	48.1	45.3	46.6	45.7	45.1	46.0	44.6	40.7
5–9	31.9	32.7	35.4	33.6	34.5	33.4	33.1	33.1	33.2
10–17	19.2	19.2	19.3	19.8	19.8	21.5	21.0	22.3	26.1
**Race and ethnicity,^c^ %**									
NH White	31.9	29.8	30.1	29.1	28.3	27.7	28.7	28.3	29.3
NH Black	32.9	33.6	34.0	34.6	35.0	35.0	33.9	34.5	36.2
Hispanic	20.7	21.5	20.8	21.5	21.2	22.0	22.3	23.3	20.1
NH Asian	2.6	2.5	2.5	2.7	2.8	3.3	3.4	3.4	2.5
NH Native American	0.7	0.7	0.8	0.9	0.8	0.8	0.8	0.8	0.9
Other^d^	4.9	5.1	5.1	4.9	5.3	5.9	6.2	5.8	6.1
Missing/invalid	6.3	6.7	6.8	6.2	6.6	5.4	4.8	3.9	4.9

Abbreviation: NH, non-Hispanic.

a Defined as aged ≤17 years.

b The *International Classification of Diseases, 9th Revision, Clinical Modification* was used through the end of September 2015; starting in October 2015, the *International Classification of Diseases, 10th Revision, Clinical Modification* was used.

c Racial and ethnic categories as they appear in the National Inpatient Sample.

d The National Inpatient Sample does not define the Other racial and ethnic category.

Hospitalization prevalence was consistently higher for male than for female children, although both groups experienced a decline in hospitalization rate over both halves of the analysis (Table 2). All age groups also experienced declines in hospitalization rates. Rates were consistently highest among children aged 0 to 4 years, with lower rates for each successive age group. Non-Hispanic Black children consistently had the highest hospitalization rate, followed by children categorized in the Other racial and ethnic group. In almost all analysis years, rates were lowest among non-Hispanic Asian children and second lowest among non-Hispanic White children, although in 2017, 2018, and 2019 non-Hispanic White children had the lowest prevalence. Hospitalization rates among Hispanic children and non-Hispanic Native American children were similar to one another, being higher than those for non-Hispanic White and non-Hispanic Asian children, but lower than rates among non-Hispanic Black children and children categorized in the Other racial and ethnic group. The Figure shows hospitalization rates by race and ethnicity over both halves of the analysis, as well as for 2020. Overall, hospitalization rates for all groups decreased over both halves of the analysis, and there was a sharp decrease in rates for all racial and ethnic groups between 2019 and 2020.

**Table 2:** Estimates of Population-Based Rates (Per 10,000 Population) of Pediatric^a^ Asthma Hospitalizations, by Demographic Characteristic, US National Inpatient Sample, 2012–2020^b^

Characteristic	2012	2013	2014	2015^a^	2016	2017	2018	2019	2020
	% (95% CI)								
**Sex**									
Male	19.0 (16.9–21.0)	16.6 (14.7–18.5)	17.1 (15.2–19.1)	14.2 (12.6–15.8)	13.0 (11.5–14.5)	12.4 (11.0–13.9)	12.1 (10.6–13.5)	10.5 (9.3–11.8)	4.3 (3.8–4.9)
Female	11.9 (10.6–13.2)	10.7 (9.4–11.9)	10.8 (9.6–12.1)	9.4 (8.3–10.4)	8.7 (7.6–9.8)	8.1 (7.1–9.1)	8.1 (7.1–9.1)	7.0 (6.2–7.9)	3.1 (2.7–3.5)
**Age, y**									
0–4	28.0 (24.9–31.0)	24.4 (21.6–27.2)	23.5 (20.8–26.2)	20.4 (18.0–22.7)	18.4 (16.2–20.6)	17.2 (15.2–19.2)	17.3 (15.1–19.4)	14.7 (12.9–16.4)	5.7 (5.0–6.4)
5–9	17.8 (15.9–19.8)	16.0 (14.1–17.9)	17.8 (15.7–19.9)	14.3 (12.6–16.0)	13.5 (11.9–15.1)	12.5 (10.9–14.0)	12.2 (10.7–13.7)	10.6 (9.3–11.9)	4.4 (3.8–5.0)
10–17	6.6 (5.1–6.5)	5.8 (5.1–6.5)	6.0 (4.6–5.8)	5.2 (4.6–5.8)	4.8 (4.2–5.4)	4.9 (4.3–5.5)	4.7 (4.1–5.3)	4.3 (3.8–4.9)	2.1 (1.8–2.4)
**Race and ethnicity**									
NH White	9.4 (8.4–10.3)	7.8 (7.0–8.6)	8.1 (7.2–9.1)	6.7 (5.9–7.4)	6.0 (5.3–6.7)	5.6 (5.0–6.3)	5.8 (5.1–6.4)	5.0 (4.4–5.6)	2.2 (1.9–2.5)
NH Black	36.7 (31.2–42.2)	33.3 (28.0–38.6)	34.6 (29.3–39.8)	29.7 (25.2–34.2)	27.7 (23.4–32.0)	26.3 (22.1–30.4)	25.0 (20.8–29.2)	22.2 (18.6–25.8)	9.8 (8.2–11.3)
Hispanic	13.5 (11.2–15.7)	12.3 (10.2–14.3)	12.0 (10.1–13.9)	10.4 (8.7–12.1)	9.3 (7.8–10.8)	9.1 (7.6–10.5)	8.9 (7.4–10.4)	8.1 (6.8–9.4)	2.9 (2.4–3.4)
NH Asian	8.3 (6.5–10.1)	7.1 (5.1–9.1)	7.0 (4.9–9.1)	6.1 (4.6–7.6)	5.8 (4.1–7.5)	6.3 (4.5–8.1)	6.2 (4.2–8.2)	5.4 (4.1–6.7)	1.7 (1.3–2.1)
NH Native American	13.4 (9.6–17.1)	11.5 (7.7–15.3)	12.3 (8.9–15.7)	12.9 (8.7–17.1)	9.9 (7.1–12.7)	9.7 (6.4–13.0)	10.3 (7.0–13.6)	8.7 (5.7–11.8)	3.9 (2.4–5.3)
Other (≥2 races)^c^	19.7 (16.2–23.2)	17.5 (14.3–20.7)	17.4 (14.4–20.5)	13.8 (10.7–17.0)	13.6 (10.2–16.9)	13.9 (11.3–16.6)	14.2 (11.4–16.9)	11.2 (9.1–13.4)	4.9 (3.8–6.1)

Abbreviation: NH, Non-Hispanic.

a Defined as aged ≤17 years.

b The *International Classification of Diseases, 9th Revision, Clinical Modification* was used through the end of September 2015; starting in October 2015, the *International Classification of Diseases, 10th Revision, Clinical Modification* was used.

c The numerator for this group is the number of children listed as having a race of Other in the National Inpatient Sample, whereas the denominator is the number of children estimated to identify as “non-Hispanic, 2 or more races,” according to the Census Bureau’s vintage 2020 population estimates.

**Figure:**
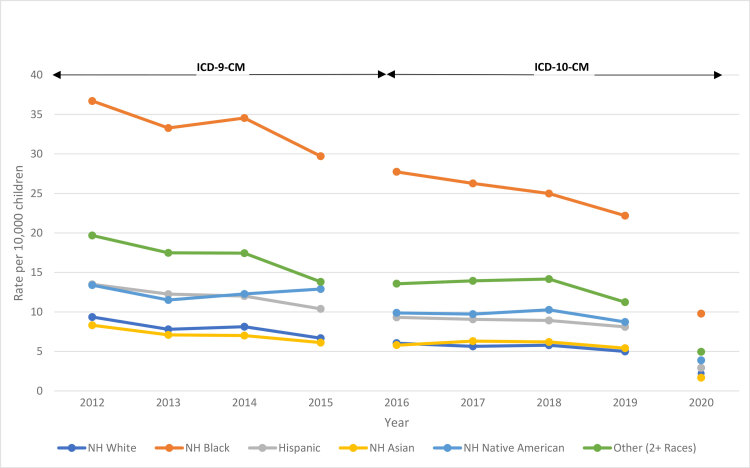
Estimated rates of pediatric asthma hospitalization, by race and ethnicity, per 10,000 population, US, 2012–2020. Data on the number of hospitalizations are from the National Inpatient Sample, and population denominators are from the Census Bureau Vintage 2020 Population Estimates. The break between 2015 and 2016 represents the change in diagnostic coding from *the International Classification of Diseases, 9th Revision, Clinical Modification* (ICD-9-CM) to the *International Classification of Diseases, 10th Revision, Clinical Modification* (ICD-10-CM). Abbreviation: NH, non-Hispanic.

**Table:** 

Race and ethnicity	2012	2013	2014	2015	2016	2017	2018	2019	2020
NH White	9.36	7.80	8.13	6.67	6.04	5.63	5.78	5.00	2.20
NH Black	36.72	33.29	34.55	29.73	27.73	26.28	25.00	22.19	9.78
Hispanic	13.50	12.25	12.01	10.39	9.30	9.06	8.93	8.10	2.91
NH Asian	8.31	7.09	7.01	6.12	5.80	6.31	6.19	5.41	1.67
NH Native American	13.39	11.50	12.27	12.90	9.87	9.72	10.27	8.73	3.87
Other (≥2 races)	19.68	17.49	17.44	13.81	13.56	13.93	14.17	11.24	4.94

Results of meta-regression models indicated patterns similar to those described above, with little heterogeneity (I ≈ 0, *P* < .90 for residual heterogeneity). From 2012 to 2015, all racial and ethnic groups except non-Hispanic Asian children had, on average, a higher hospitalization prevalence than non-Hispanic White children (Table 3). Non-Hispanic Black children had the highest average hospitalization prevalence, followed by children categorized in the Other racial and ethnic group. For this earlier period (2012–2015), the common slope was −0.83 (95% CI, −1.14 to −0.52; *P* < .001), indicating that hospitalization rates per 10,000 children decreased by about 0.83 per year (Table 3). While the addition of a race–year interaction term suggested that race-specific trends may vary modestly across racial and ethnic group, overall, the results were consistent with a similar decrease across all racial and ethnic groups, based on Bayesian Information Criterion. Table 4 contains the results of the model with the race–year interaction term. The BIC of the simpler model was 89.18, whereas the BIC of the model with interaction was 101.38.

**Table 3:** Meta-Regression Results for Model Without Race by Year Interaction, US National Inpatient Sample, 2012–2015 and 2016–2019

Coefficient	2012–2015^a^		2016–2019^b^	
	Estimate (95% CI)	*P* value	Estimate (95% CI)	*P* value
Intercept	9.19 (8.54 to 9.83)	<.001	6.13 (5.64 to 6.62)	<.001
Year, slope	−0.83 (−1.14 to −0.52)	<.001	−0.35 (−0.58 to −0.12)	.003
Non-Hispanic White	1 [Reference]			
Non-Hispanic Black	25.37 (22.78 to 27.96)	<.001	19.47 (17.43 to 21.50)	<.001
Hispanic	4.05 (2.99 to 5.11)	<.001	3.23 (2.44 to 4.01)	<.001
Non-Hispanic Asian	−0.78 (−1.77 to 0.22)	.13	0.29 (−0.58 to 1.16)	.52
Non-Hispanic Native American	4.51 (7.44 to 10.76)	<.001	4.00 (2.43 to 5.58)	<.001
Other (≥2 races)	9.10 (7.44 to 10.76)	<.001	7.42 (6.07 to 8.77)	<.001

Abbreviation: BIC, Bayesian Information Criterion.

a BIC = 89.18.

b BIC = 76.90.

**Table 4:** Meta-Regression Results for Model With Race by Year Interaction, US National Inpatient Sample, 2012–2015 and 2016–2019

	2012–2015^a^		2016–2019^b^	
Coefficients	Estimate (95% CI)	*P* value	Estimate (95% CI)	*P* value
Intercept	9.11 (8.35 to 9.88)	<.001	6.06 (5.50 to 6.62)	<.001
Year, slope	−0.78 (−1.16 to −0.40)	<.001	−0.31 (−0.59 to −0.02)	.03
**Race**				
NH White	1 [Reference]			
NH Black	27.47 (22.90 to 32.04)	<.001	21.96 (18.39 to 25.53)	<.001
Hispanic	4.39 (2.44 to 6.34)	<.001	3.35 (2.01 to 4.70)	<.001
NH Asian	−0.96 (−2.67 to 0.77)	.28	0.05 (−1.46 to 1.56)	.95
NH Native American	3.54 (0.29 to 6.78)	.03	3.98 (1.50 to 6.47)	.002
Other (≥2 races)	10.67 (7.73 to 13.62)	<.001	8.50 (5.88 to 11.11)	<.001
**Race by year interaction**				
Year × NH White	1 [Reference]			
Year × NH Black	−1.27 (−3.54 to 1.01)	.28	−1.52 (−3.31 to 0.27)	.10
Year × Hispanic	−0.20 (−1.15 to 0.75)	.68	−0.08 (−0.77 to 0.61)	.82
Year × NH Asian	−0.10 (−0.73 to 0.94)	.81	0.14 (−0.59 to 0.86)	.72
Year × NH Native American	0.67 (−1.11 to 2.45)	.46	0.02 (−1.33 to 1.37)	.98
Year × Other (≥2 races)	−1.00 (−2.51 to 0.54)	.20	−0.59 (−1.82 to 0.64)	.34

Abbreviations: BIC, Bayesian Information Criterion; NH, non-Hispanic.

a BIC = 101.38.

b BIC = 88.92.

Results for the period 2016–2019 were similar to those generated for 2012–2015. All racial and ethnic groups except for non-Hispanic Asian children had, on average, a higher hospitalization prevalence than non-Hispanic White children, regardless of model complexity, although the race-specific coefficients were lower than in 2012–2015 (Table 3). Non-Hispanic Black children still had the highest average prevalence, followed by children categorized in the Other racial and ethnic group. For this later period (2016–2019), the magnitude of the slope was lower (−0.35; 95% CI, −0.58 to −0.12; *P* = .003), but still suggested a decreasing temporal trend of the hospitalization rate per 10,000 children (Table 3). The addition of the race–year interaction term did not provide evidence of variation in race-specific trends; CIs for every race–year interaction term included the null, and the likelihood ratio test was not significant (*P* > .05) for years 2012–2015 and for years 2016–2019. Once again, the BIC strongly favored the simpler model with a common slope for all racial and ethnic groups. Table 4 contains the results of the model with the race–year interaction term; the BIC of the simpler model was 76.90, whereas the BIC of the interaction model was 88.92.

## Discussion

Overall, the number of pediatric asthma hospitalizations decreased during both halves of the analysis. In addition, the hospitalization rates for each racial and ethnic group individually decreased during both halves of the analysis. However, no evidence exists that rates for any racial and ethnic group declined faster (or slower) than for non-Hispanic White children. Thus, although the rate differences between the asthma hospitalization rates of non-Hispanic White children and those of non-Hispanic Black children and children categorized in the Other racial and ethnic group are decreasing, the pre-existing disparities, as defined by the rate ratio, are not decreasing. Therefore, the long-documented racial and ethnic disparities in asthma outcomes persist. These results are similar to the findings of Akinbami et al (6), who reported similar rates of decline in population-based rates of asthma hospitalizations among both White and Black children between 2001 and 2010, indicating the Black/White racial disparity had not improved despite overall improvements in the number of hospitalizations. Future analyses expounding on other health disparity measures, including relative changes, can add to our understanding of changes in asthma-related health disparities.

Several potential factors may explain why the overall number and prevalence of hospitalizations declined over the analysis period. Management of asthma exacerbations in emergency medicine settings has improved in the last decade (18), potentially leading to fewer inpatient admissions from EDs. Similarly, improved access to outpatient care may have boosted provider and parent confidence in the ability to manage asthma in outpatient settings, also leading to fewer inpatient admissions from EDs. The cost for pediatric inpatient stays has also increased substantially in the past 2 decades (19), which has perhaps led to reluctance to use inpatient care, especially given the aforementioned improvements in emergency and outpatient care.

Because racial and ethnic asthma disparities have persisted over time, opportunities to use frameworks that consider the unique needs of people from disproportionately affected racial and ethnic minority groups will continue. Many frameworks exist that aim to address health disparities and achieve health equity. Potentially promising frameworks include the Social Determinants of Health (20) (1 of 3 priority areas for Healthy People 2030) and Vital Conditions for Health and Wellbeing (21). Both frameworks address nonmedical factors that nonetheless influence health outcomes and whose negative consequences are most frequently borne by socially disadvantaged groups. One framework specific to asthma is the National Asthma Control Program’s EXHALE Technical Package, which emphasizes not only individual-level factors in asthma control, but also the broader environmental conditions that contribute to adverse asthma outcomes (22).

Anchoring future asthma interventions in these and other frameworks that address the specific needs of populations who face greater social disadvantage may help address the existing racial and ethnic asthma disparities. Both individual- and community-level interventions have a role to play. On the individual level, interventions promoting access and engagement with primary care and more consistent use of controller medications have been shown to decrease asthma-related ED visits and hospitalizations (23). Community-based interventions also have a proven impact, such the Children’s Hospital of Philadelphia CAPP+ program, which demonstrated that addressing social determinants of health (in this case, housing quality) reduces emergency health care utilization among children with asthma (24). Ultimately, achieving racial and ethnic equity in asthma outcomes will likely require interventions focused on both individual- and community-level determinants that are specifically tailored to the needs of individuals from racial and ethnic groups that are disproportionately affected.

Race and ethnicity are not in and of themselves the main drivers of asthma disparities. Rather, they are markers of other factors, such as socioeconomic status and experiences of interpersonal and structural discrimination, that more directly influence health disparities (25). For example, material hardship and poor housing quality may partially mediate the relationship between race and asthma ED visits (26). Environmental exposures, as well as cultural and psychosocial experiences, that vary across racial and ethnic groups may also play a role in the risk of adverse asthma outcomes (27–29). However, such factors are not inherent in certain racial and ethnic groups per se, but rather reflect larger systems of inequity.

### Limitations

Our analyses were limited by the structure and completeness of the NIS. The NIS does not include data from all US states and does not include any data from US territories. However, in every year under study, HCUP partners participating in the NIS collectively covered 98% of the US population, resulting in a nationally representative sample despite the nonparticipating states. One potential source of bias stems from the nonstandardized collection of racial and ethnic status in the NIS. Collection and reporting of racial and ethnic status vary by and within HCUP partners. While some facilities routinely ask patients to self-identify, others may base racial and ethnic status collection on providers’ judgement. However, because experiences of interpersonal and structural injustice (which would be primarily based on externally perceived racial and ethnic status) are some of the main factors contributing to racial and ethnic health disparities (25), we assumed this bias had only a negligible effect on our results. There may also be a disconnect in the numerator and population denominators when assigned racial and ethnic status does not match an individual’s self-identification in the Census. Still, these facts, along with the differences in race and ethnicity reporting categories between the NIS and the Census Bureau Vintage 2020, likely resulted in some degree of misclassification. We believe these biases are nondifferential, especially for the largest racial and ethnic groups with a direct match between the NIS and the Census Bureau figures (non-Hispanic White, non-Hispanic Black, and Hispanic). Misclassification likely had the largest effect in the Other racial and ethnic group, as the population denominator used (individuals identifying in the Census as belonging to 2 or more racial groups) is not directly comparable. Another potential source of downward bias in all estimates is the incomplete collection of racial and ethnic status and subsequent exclusion of children with a missing racial and ethnic status. This exclusion may also have biased the estimated disparities if missingness is differential by racial and ethnic group, with the greatest potential for bias in estimates for the smaller racial and ethnic groups. Although between 3% and 7% of children in our data set had no identified racial and ethnic status, depending on year, this estimate is substantially improved from those of prior research (4). Given the small and relatively stable percentage of children with missing racial and ethnic status, we do not believe the exclusion of this group had a substantial impact on our results. There is also potential masking resulting from the NIS categorization of Hispanic ethnicity without consideration of Hispanic subgroup. Differences in asthma prevalence between various Hispanic subgroups has been well documented, with Puerto Rican children in particular bearing a disproportionate asthma burden (30). This lack of data into Hispanic patients’ ethnic subgroup is likely masking important differences in asthma hospitalizations among Hispanic subpopulations.

Another set of limitations concerns changes in diagnostic coding and health care utilization patterns. Between the third and fourth calendar quarters of 2015, diagnostic coding of health care encounters changed from ICD-9-CM to ICD-10-CM. Relevant to this analysis, this change involved the differentiation of several chronic respiratory illnesses, such as chronic obstructive pulmonary disease, previously classified under the same set of codes as asthma. However, because the newly differentiated illnesses primarily affect adult populations, as well as the lack of dramatic shift in the demographic characteristics of hospitalized children (Table 1), the effect of these coding changes on pediatric populations is likely negligible. Still, we opted to calculate trends before and after this coding change separately to reduce the possibility of bias. Health care utilization shifted dramatically between 2019 and 2020 due to the onset of the COVID-19 pandemic. As AHRQ notes, the overall number of discharges in 2020 decreased by almost 9% compared with 2019 (31). Other authors identified shifts in health care utilization among patients with asthma specifically, noting a sharp decline in asthma-related ED visits in 2020, even more so than for ED visits overall (11,12,32,33). We therefore excluded 2020 from our trend analyses. As more recent years of the NIS are published and used to quantify the impact of the COVID-19 pandemic on health care utilization, future research will be better poised to analyze trends spanning the pandemic years.

### Conclusion

Non-Hispanic Black and children categorized in the Other racial and ethnic group remain disproportionately burdened by asthma-related hospitalizations, as they have been for decades. Overall, the prevalence of pediatric asthma hospitalizations is decreasing, and prevalence is also decreasing among all racial and ethnic groups individually, albeit at a similar rate among all groups. While the overall decline is a positive development, the similar rates of decline among all groups has meant the continued persistence of racial and ethnic disparities. As such, interventions based on frameworks that address the unique challenges encountered by groups who face greater social disadvantage may be useful in reducing racial and ethnic asthma disparities.

## References

[1] Centers for Disease Control and Prevention, National Center for Environmental Health. About asthma. Updated June 1, 2023. Accessed January 24, 2024. https://www.cdc.gov/asthma/about/

[2] Centers for Disease Control and Prevention, National Center for Health Statistics. Percentage of current asthma for children under age 18, United States, 2021. National Health Interview Survey. Accessed June 24, 2024. https://wwwn.cdc.gov/nhisdataquerytool/SHS_child/index.html

[3] Montalbano L , Ferrante G , Montella S , Cilluffo G , Di Marco A , Bozzetto S , ; Italian Pediatric Severe Asthma Network (IPSAN) Program of Italian Paediatric Respiratory Society (IPRS). Relationship between quality of life and behavioural disorders in children with persistent asthma: a Multiple Indicators Multiple Causes (MIMIC) model. *Sci Rep.* 2020;10(1):6957. 10.1038/s41598-020-62264-9 32332757 PMC7181655

[4] Hamelmann E , von Mutius E , Bush A , Szefler SJ . Addressing the risk domain in the long-term management of pediatric asthma. *Pediatr Allergy Immunol.* 2020;31(3):233–242. 10.1111/pai.13175 31732983 PMC7217022

[5] Moorman JE , Rudd RA , Johnson CA , King M , Minor P , Bailey C , ; Centers for Disease Control and Prevention (CDC). National surveillance for asthma — United States, 1980–2004. *MMWR Surveill Summ.* 2007;56(8):1–54. 17947969

[6] Akinbami LJ , Moorman JE , Simon AE , Schoendorf KC . Trends in racial disparities for asthma outcomes among children 0 to 17 years, 2001–2010. *J Allergy Clin Immunol.* 2014;134(3):547–553.e5. 10.1016/j.jaci.2014.05.037 25091437 PMC4757843

[7] Healthcare Cost and Utilization Project. HCUP National Inpatient Sample. Agency for Healthcare Research and Quality. Accessed June 24, 2024. https://www.hcup-us.ahrq.gov/nisoverview.jsp 21413206

[8] US Census Bureau Population Division. Monthly population estimates by age, sex, race, and Hispanic origin for the United States: April 1, 2010, to July 1, 2020. Washington (DC): US Government Printing Office; 2021.

[9] US Census Bureau. Methodology for the United States population estimates: vintage 2020, version 2; May 2021. Accessed June 24, 2024. https://www2.census.gov/programs-surveys/popest/technical-documentation/methodology/2010-2020/methods-statement-v2020-final.pdf

[10] Akinbami LJ , Simon AE , Rossen LM . Changing trends in asthma prevalence among children. *Pediatrics.* 2016;137(1):1–7. 10.1542/peds.2015-2354 26712860 PMC4755484

[11] Office of Management and Budget. Revisions to the standards for the classification of Federal data on race and ethnicity. *Fed Regist* *.* 1997;62(210):58782–90.

[12] US Census Bureau Population Division. Documentation for vintage 2020 bridged-race postcensal population estimates for calculating vital rates. Washington (DC): US Government Printing Office; 2021.

[13] Taquechel K , Diwadkar AR , Sayed S , Dudley JW , Grundmeier RW , Kenyon CC , . Pediatric asthma health care utilization, viral testing, and air pollution changes during the COVID-19 pandemic. *J Allergy Clin Immunol Pract.* 2020;8(10):3378–3387.e11. 10.1016/j.jaip.2020.07.057 32827728 PMC7438361

[14] Hurst JH , Zhao C , Fitzpatrick NS , Goldstein BA , Lang JE . Reduced pediatric urgent asthma utilization and exacerbations during the COVID-19 pandemic. *Pediatr Pulmonol.* 2021;56(10):3166–3173. 10.1002/ppul.25578 34289526 PMC8441648

[15] Gasparrini A , Armstrong B , Kenward MG . Multivariate meta-analysis for non-linear and other multi-parameter associations. *Stat Med.* 2012;31(29):3821–3839. 10.1002/sim.5471 22807043 PMC3546395

[16] Sera F , Armstrong B , Blangiardo M , Gasparrini A . An extended mixed-effects framework for meta-analysis. *Stat Med.* 2019;38(29):5429–5444. 10.1002/sim.8362 31647135

[17] R Core Team. R: a language and environment for statistical computing. R Foundation for Statistical Computing; 2023. Accessed June 24, 2024. https://www.R-project.org/

[18] Rogerson C , He T , Rowan C , Tu W , Mendonca E . Ten-year trends in hospital encounters for pediatric asthma: an Indiana experience. *J Asthma.* 2022;59(12):2421–2430. 10.1080/02770903.2021.2010750 34818967

[19] Short HL , Sarda S , Travers C , Hockenberry J , McCarthy I , Raval MV . Pediatric inpatient-status volume and cost at children’s and nonchildren’s hospitals in the United States: 2000–2009. *Hosp Pediatr.* 2018;8(12):753–760. 10.1542/hpeds.2017-0152 30409769

[20] Hacker K , Auerbach J , Ikeda R , Philip C , Houry D . Social determinants of health — an approach taken at CDC. *J Public Health Manag Pract.* 2022;28(6):589–594. 10.1097/PHH.0000000000001626 36194813 PMC9555578

[21] Community Commons. Seven vital conditions for health and well-being. Accessed June 24, 2024. https://www.communitycommons.org/collections/Seven-Vital-Conditions-for-Health-and-Well-Being

[22] Hsu J , Sircar K , Herman E , Garbe P . EXHALE: a technical package to control asthma. Atlanta (GA): National Center for Environmental Health, Centers for Disease Control and Prevention; 2018.

[23] Kim Y , Pirritano M , Parrish KM . Determinants of racial and ethnic disparities in utilization of hospital-based care for asthma among Medi-Cal children in Los Angeles. *J Asthma.* 2022;59(8):1521–1530. 10.1080/02770903.2021.1955131 34252345

[24] Strane D , Flaherty C , Kellom K , Kenyon CC , Bryant-Stephens T . A health system–initiated intervention to remediate homes of children with asthma. *Pediatrics.* 2023;151(5):e2022058351. 10.1542/peds.2022-058351 37042200

[25] Phelan JC , Link BG . Is racism a fundamental cause of inequalities in health? *Annu Rev Sociol.* 2015;41(1):311–330. 10.1146/annurev-soc-073014-112305

[26] Hughes HK , Matsui EC , Tschudy MM , Pollack CE , Keet CA . Pediatric asthma health disparities: race, hardship, housing, and asthma in a national survey. *Acad Pediatr.* 2017;17(2):127–134. 10.1016/j.acap.2016.11.011 27876585 PMC5337434

[27] Drake KA , Galanter JM , Burchard EG . Race, ethnicity and social class and the complex etiologies of asthma. *Pharmacogenomics.* 2008;9(4):453–462. 10.2217/14622416.9.4.453 18384258 PMC2746736

[28] Landeo-Gutierrez J , Forno E , Miller GE , Celedón JC . Exposure to violence, psychosocial stress, and asthma. *Am J Respir Crit Care Med.* 2020;201(8):917–922. 10.1164/rccm.201905-1073PP 31801032 PMC7159436

[29] Moraes TJ , Sears MR , Subbarao P . Epidemiology of asthma and influence of ethnicity. *Semin Respir Crit Care Med.* 2018;39(1):3–11. 10.1055/s-0037-1618568 29427980

[30] Lara M , Akinbami L , Flores G , Morgenstern H . Heterogeneity of childhood asthma among Hispanic children: Puerto Rican children bear a disproportionate burden. *Pediatrics.* 2006;117(1):43–53. 10.1542/peds.2004-1714 16396859

[31] Agency for Healthcare Research and Quality. Introduction to the HCUP National Inpatient Sample (NIS) 2020; 2022. Accessed June 24, 2024. https://hcup-us.ahrq.gov/db/nation/nis/NISIntroduction2020.pdf

[32] Gaffney A , Himmelstein DU , Woolhandler S . Population-level trends in asthma and chronic obstructive pulmonary disease emergency department visits and hospitalizations before and during the Coronavirus disease 2019 pandemic in the United States. *Ann Allergy Asthma Immunol.* 2023;131(6):737–744. 37619778 10.1016/j.anai.2023.08.016

[33] Ye D , Gates A , Radhakrishnan L , Mirabelli MC , Flanders WD , Sircar K . Changes in asthma emergency department visits in the United States during the COVID-19 pandemic. *J Asthma.* 2023;60(8):1601–1607. 10.1080/02770903.2023.2165445 36608267 PMC10293019

